# Unveiling Macrophage Content as a Predictive Biomarker for Intraoperative ICG Imaging Efficacy in Lung Cancer

**DOI:** 10.1002/advs.202504498

**Published:** 2025-08-04

**Authors:** Yue Yan, Jiahui Mi, Yun Li, Guanchao Jiang, Yimeng Zhang, Yanguo Liu, Hui Zhao, Jianfeng Li, Xing Yang, Jun Wang, Fan Yang, Kezhong Chen, Yiguang Wang, Jian Zhou

**Affiliations:** ^1^ Department of Central Laboratory Peking University First Hospital Beijing 100034 China; ^2^ Department of Thoracic Surgery Peking University People's Hospital Beijing 100044 China; ^3^ Thoracic Oncology Institute Peking University People's Hospital Beijing 100044 China; ^4^ State Key Laboratory of Natural and Biomimetic Drugs Peking University Beijing 100191 China; ^5^ Department of Thoracic Surgery Peking University People's Hospital Qingdao 266111 China; ^6^ Department of Nuclear Medicine Peking University People's Hospital Beijing 100044 China; ^7^ Beijing Key Laboratory of Molecular Pharmaceutics and New Drug Delivery System School of Pharmaceutical Sciences Peking University Beijing 100191 China

**Keywords:** ICG imaging outcomes, inter‐patient variability, lung cancer, macrophages, predictive biomarker

## Abstract

Due to the phenotypic and genotypic heterogeneity of tumors, the efficacy of intraoperative indocyanine green (ICG) imaging in lung cancer exhibits significant inter‐patient variability. This study identifies macrophage content as a critical predictive biomarker for ICG imaging outcomes, offering both mechanistic, and clinical insights into this variability. Mechanistically, macrophages are demonstrated to serve as the principal ICG reservoirs in tumor tissues, exhibiting seven‐fold higher uptake capacity compared to cancer cells. This critical role is confirmed by significantly diminished ICG accumulation following macrophage depletion in patient‐derived xenograft (PDX) models. Clinically, a strong correlation is observed between imaging quality and macrophage content, with solid nodules exhibiting superior ICG uptake compared to ground‐glass nodules due to higher macrophage infiltration. Furthermore, the strong tumor‐to‐normal ratio (TNR) association with preoperative maximum Standardized Uptake Value (SUVmax) on PET‐CT suggests the feasibility of predicting ICG‐guided surgery outcomes through routine imaging. The substantial contribution of macrophages to this predictive capability is a significant discovery, offering a novel biomarker for patient stratification in ICG‐guided surgery. These insights not only deepen our comprehension of the intricate interplay between ICG and the lung cancer microenvironment but also open new avenues for the development of more personalized and precise surgical strategies.

## Graphical abstract

Indocyanine Green (ICG)‐guided lung cancer surgery efficacy varies significantly among patients. This study identifies tumor macrophage content as a predictive biomarker. Macrophages are primary ICG reservoirs, storing seven‐fold more ICG than cancer cells. Clinically, solid nodules absorb ICG better than ground‐glass nodules due to their higher macrophage content. Preoperative macrophage assessment enables patient selection for personalized surgical optimization.

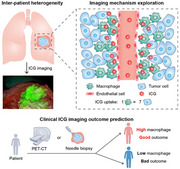

## 1 Introduction

Lung cancer remains the leading cause of cancer‐related mortality worldwide, with surgical resection being a cornerstone of curative treatment.^[^ ^1^ ^]^ Therefore, accurate intraoperative identification of tumor margins is critical for achieving complete resection and improving patient outcomes.^[^ ^2^ ^]^ However, conventional imaging modalities, such as computed tomography (CT) and positron emission tomography (PET), while useful for preoperative planning,^[^ ^3^, ^4^ ^]^ lack the real‐time precision required for intraoperative guidance.^[^ ^5^ ^]^ Near‐infrared (NIR) fluorescence imaging, with its high spatial resolution capabilities,^[^ ^6^ ^]^ has emerged as a promising tool for real‐time tumor localization during lung cancer surgery.^[^ ^7^ ^]^ Among the available NIR fluorophores, the US Food and Drug Administration (FDA) approved indocyanine green (ICG) is the most widely used due to its excellent safety profile and deep tissue penetration.^[^ ^8^ ^]^ ICG, a water‐soluble amphiphilic molecule,^[^ ^9^ ^]^ binds to serum proteins such as albumin upon intravenous administration to form stable complexes,^[^ ^10^ ^]^ enabling its accumulation in tumors through the enhanced permeability and retention (EPR) effect. This property has made ICG a valuable tool for intraoperative tumor localization,^[^ ^11^ ^]^ particularly in lung cancer surgery, where it has been shown to improve the detection of small nodules and intersegmental planes.^[^ ^12^ ^]^ However, despite its potential, the efficacy of ICG imaging varies significantly among patients, highlighting the need to better understand the underlying mechanisms driving ICG accumulation in lung cancer tissues.

Currently, the mechanisms governing ICG fluorescence imaging in lung cancer remain poorly understood. While the EPR effect is widely acknowledged as the primary driver of ICG accumulation in tumors, significant interpatient variability in imaging efficacy persists.^[^ ^13^ ^]^ This clinical inconsistency suggests that tumor microenvironmental factors beyond passive vascular leakage—particularly heterogeneous cellular composition and metabolic dynamics—may critically influence ICG distribution and retention.^[^ ^14^ ^]^ Compounding this complexity, the lung cancer microenvironment features intricate cellular crosstalk among malignant cells, tumor‐associated macrophages, stromal components, and immune infiltrates, collectively shaping both tumor biology and therapeutic responses.^[^ ^15^ ^]^ While existing studies in oncological imaging have primarily focused on ICG's physicochemical interactions with tumor cells,^[^ ^11^, ^16^ ^]^ emerging evidence from non‐oncological contexts hints at ICG's affinity for other specific cell populations. For instance, studies in atherosclerosis demonstrate that ICG selectively targets lipid‐rich macrophages within plaques.^[^ ^17^ ^]^ This observed pathogenetic targeting prompts critical inquiries into whether parallel biological interactions mediate ICG uptake in lung cancer. However, current research has yet to delineate ICG's spatial distribution patterns within lung tumors or clarify its cellular targets. This knowledge gap hinders the optimization of ICG‐guided surgery, as evidenced by variable tumor‐to‐background ratios across patients. Therefore, the mechanisms underlying its role in intraoperative fluorescence imaging for lung cancer remain largely unknown. For instance, the specific distribution characteristics of ICG in lung cancer tissues, its interactions with different cell types in the tumor microenvironment, and its real‐time imaging performance during surgery all require further in‐depth research. Additionally, the effectiveness of ICG in different types of tumors may vary, so personalized application strategies for specific tumor types are also worth further exploration.

Herein, we systematically investigate the fluorescence imaging mechanism of ICG in lung cancer through clinical and preclinical studies. Our multimodal analysis reveals macrophages as the principal ICG reservoirs in human lung cancer tissues, exhibiting sevenfold greater ICG uptake compared to tumor cells. In addition, experiments using patient‐derived xenograft (PDX) models establish a causal relationship between macrophage depletion and diminished ICG accumulation, demonstrating that macrophage content may be a significant factor in determining imaging contrast. Our research also broadens to assess the distribution of macrophages and ICG uptake across different pathological types of clinical lung cancer. These clinical observations further reveal that solid nodules with high macrophage infiltration achieve superior ICG visualization compared to ground‐glass nodules. Moreover, we've uncovered a significant link between the Tumor‐to‐Normal Ratio (TNR) and the maximum Standardized Uptake Value (SUVmax) from ^18^F‐FDG‐PET/CT scans, suggesting a predictive potential for the success of ICG imaging prior to surgery, heavily reliant on the substantial contribution of macrophages. These findings collectively enhance our comprehension of ICG's interaction with the lung cancer microenvironment, particularly highlighting the role of macrophages as a novel biomarker for predicting ICG imaging outcomes. This research not only provides a new perspective on the imaging mechanism of ICG but also offers a practical tool for clinicians to select patients who are most likely to benefit from ICG‐guided surgery, thereby improving the precision and personalization of lung cancer treatment.

## 2 Results

### 2.1 ICG Co‐Localizes with Lung Carbon Particles

**Figure 1:**
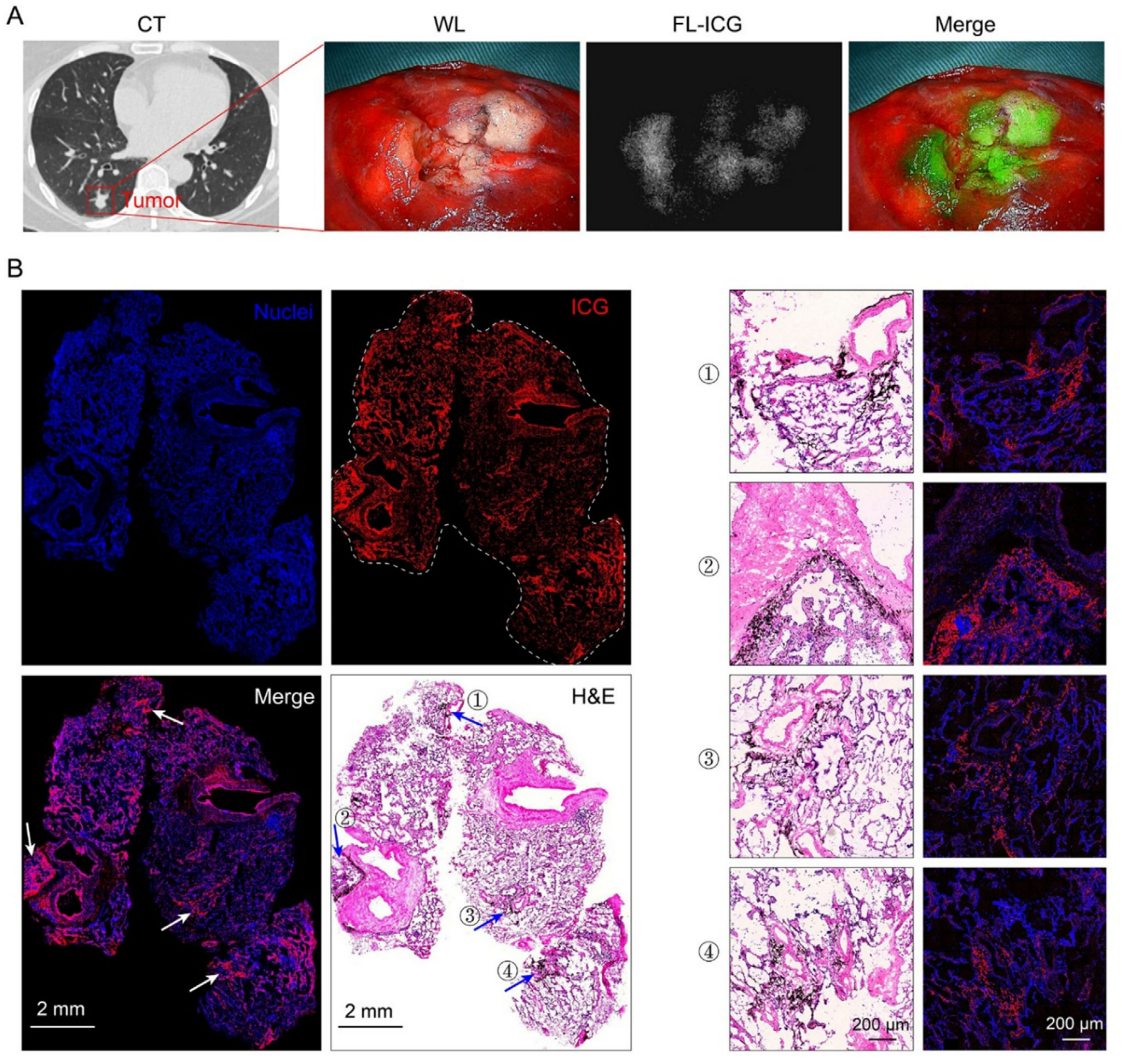
Near‐infrared imaging and pathological tissue analysis of lung cancer samples. A) Computed tomography, visible light image, and fluorescence image of lung cancer. B) Fluorescence and H&E staining scanning images of the patient lung cancer sample sections. In the fluorescence scanning image, the cell nuclei and ICG were depicted in blue and red pseudo‐colors, respectively. The ICG distribution within the fluorescence image exhibited excellent co‐localization with the carbon particles visible in the H&E staining image. Four representative regions were indicated by arrows, and the synchronized enlarged images of the fluorescence and H&E images were shown on the right panel.

Based on the preoperative CT scan data of the patient for tumor mapping, we pinpointed the tumor's location and administered ICG to the patient 24 h before surgery to aid in intraoperative fluorescence imaging. During the surgical procedure, a near‐infrared thoracoscope system was employed to assess the imaging effect of ICG. The findings revealed a substantial concentration of ICG within the tumor area, notably surpassing that of the adjacent healthy lung tissue, demonstrating excellent imaging quality, which ensured precise tumor localization and accurate determination of surgical margins (**Figure** **1**A).

Upon examination of the H&E‐stained lung cancer sample, we observed an abundance of black carbon particles, likely attributed to factors such as long‐term exposure to tobacco smoke or environmental pollution. Interestingly, the ICG fluorescence distribution in the fluorescent imaging sections showed a high degree of spatial correlation with the carbon particle distribution observed in the H&E staining (Figure 1B). To further clarify this phenomenon, we identified four areas with a high concentration of carbon particles in the H&E staining and synchronized their magnification with the fluorescent imaging sections. This meticulous alignment allowed for a clearer observation of the consistency between the distribution patterns of carbon particles and ICG fluorescence.

### 2.2 Macrophage‐Driven ICG Enrichment in Lung Cancers

**Figure 2:**
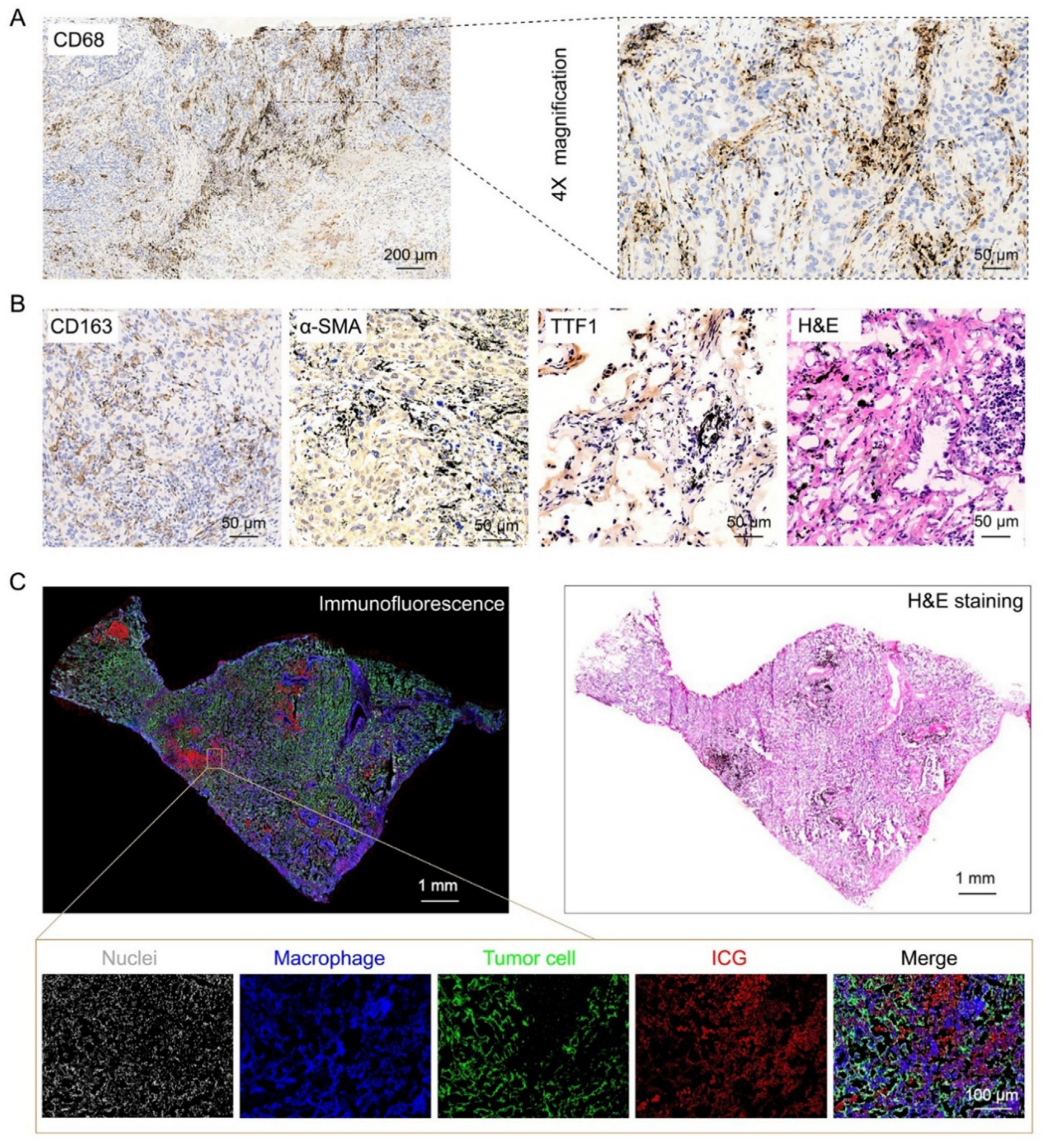
The distribution of ICG and carbon particles among various cells in lung cancer samples. A,B) Immunohistochemical staining of macrophages (CD68, CD163), fibroblasts (α‐SMA), and tumor cells (TTF1) in lung cancer tissue samples to investigate the distribution of carbon particles among these cells. C) The good co‐localization between ICG and macrophages. Full‐slide scanning of the lung cancer tissue sections that were immunofluorescently stained with tumor and macrophage cells. The nuclei, macrophages, tumor cells, and ICG were displayed in gray, blue, green, and red pseudo‐color, respectively.

Based on the aforementioned spatial co‐localization phenomenon, we focused on the cellular localization analysis of carbon particles to elucidate the biological basis for the enrichment of ICG in tumor tissues. Specifically, we performed immunohistochemical experiments to identify the associated cell types. The findings revealed a significant co‐localization of carbon particles with macrophages, in stark contrast to the lack of association with fibroblasts and tumor cells (**Figure** **2**A,B). This observation prompted us to conclude that ICG, similar to carbon particles, is predominantly taken up by macrophages in lung cancer tissues. To further substantiate this conclusion, we employed immunofluorescence techniques to concurrently label tumor cells and macrophages in lung cancer samples, thereby scrutinizing the distribution pattern of ICG. The results from immunofluorescence provided a clear and compelling visual confirmation of our initial findings. The red pseudocolored ICG was observed to be in close proximity to the blue pseudocolored macrophages, indicating a strong co‐localization. In contrast, there was a relatively lower amount of ICG fluorescence co‐localizing with the green pseudocolored tumor cells (Figure 2C). These findings not only more intuitively verified our above conclusion but also offer a visual representation of the selective interaction between ICG and macrophages within the complex tumor microenvironment.

### 2.3 Dissecting the Contribution of Macrophages and Tumor Cells on ICG Imaging

**Figure 3:**
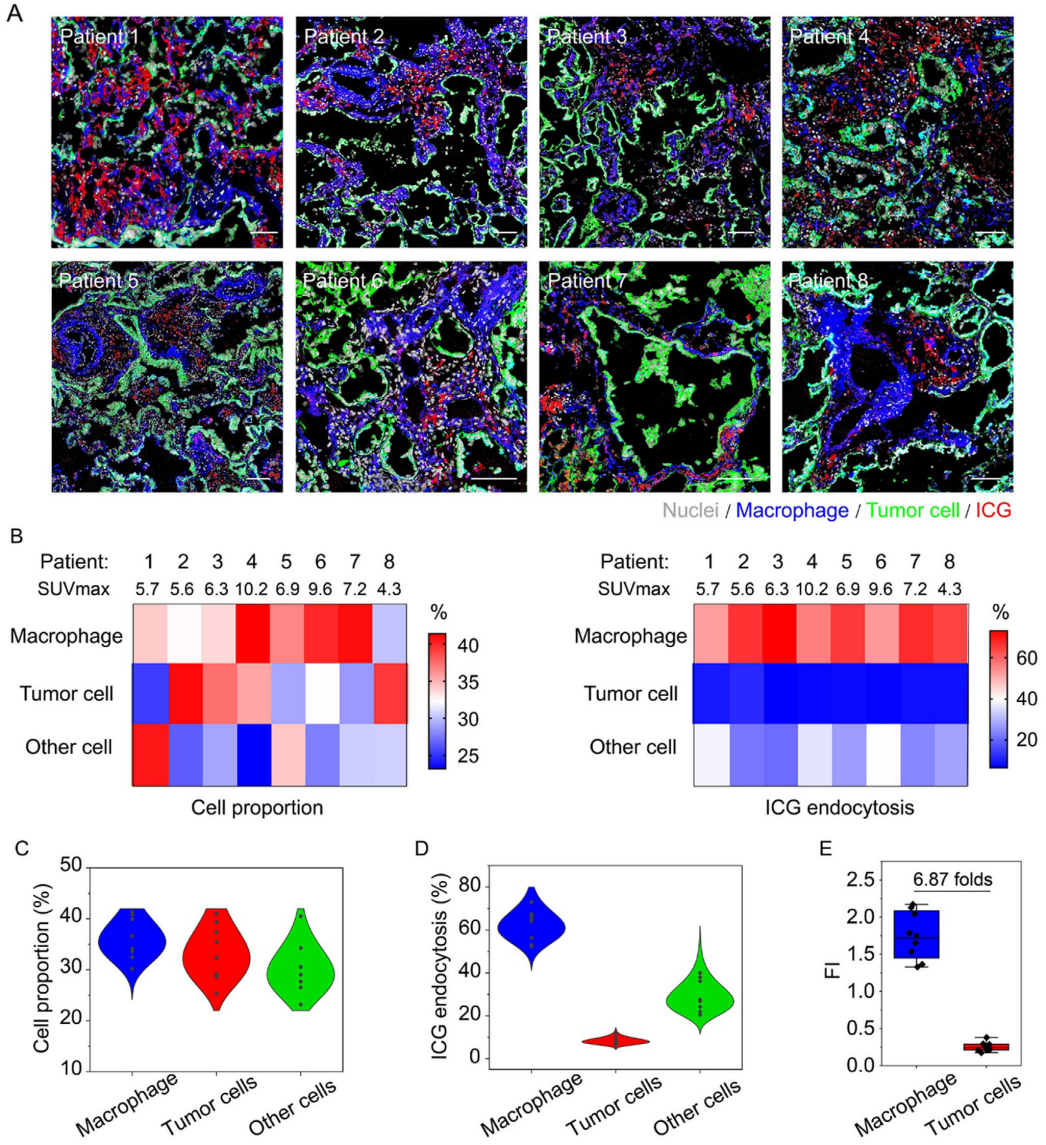
Dissecting the contribution of macrophages and tumor cells on ICG imaging. A) Immunofluorescence staining of 8 lung cancer patient samples. The nuclei, macrophages, tumor cells, and ICG were displayed in gray, blue, green, and red pseudo‐color, respectively. Scale bar = 100 µm. B) The heatmap displayed the proportion of various cell types in each lung cancer samples (left panel), as well as the percentage of ICG uptake by each cell type (right panel). C,D) Quantitative analysis of cellular composition (C) and the percentage of ICG uptake by different cell types (D) in lung cancer tissue samples of eight patients. E) The ICG uptake capability comparison of macrophages and tumor cells in lung cancer tissue. Data were presented as mean ± s.d. (*n* = 8).

The conventional understanding suggests that ICG is absorbed by the cancerous cells in lung cancer samples.^[^ ^11^, ^16^, ^18^ ^]^ In contrast, our initial findings of this study revealed a significant colocalization between ICG and macrophages, indicating a potentially overlooked role of these macrophages in the uptake and imaging of ICG. This discovery invited a reevaluation of the mechanisms behind ICG distribution within tumors. Therefore, to further elucidate the contributions of tumor cells and macrophages to the specificity of ICG intraoperative imaging, we enrolled a group of eight non‐small cell lung cancer (NSCLC) patients (**Table** **1**). The cohort consisted of three males and five females with solid lung nodules, and the mean tumor‐to‐normal tissue ratio (TNR) was 2.9. Employing immunofluorescence staining, a pronounced colocalization of ICG with macrophages was observed (**Figure** **3**A). A detailed quantitative analysis unveiled a notable heterogeneity in cellular composition and ICG distribution within the lung cancer tissues of the patients (Figure 3B,C). Specifically, the endocytosis percentages of ICG by tumor cells, macrophages, and other cells within the samples were found to be 8.35%, 62.2%, and 29.4%, respectively. It was particularly striking that macrophages accounted for the uptake of nearly two‐thirds of the ICG within the tumor tissue, underscoring their critical role in ICG imaging of lung cancer tissues (Figure 3D). Further in‐depth analysis comparing the ICG uptake efficiency of macrophages and tumor cells in lung cancer tissue revealed a compelling discovery: the ICG uptake by macrophages was about seven times higher than that by tumor cells (Figure 3E). This remarkable discrepancy emphasizes the pivotal role of macrophages in achieving high‐contrast imaging of lung cancer tissues.

**Table 1:** Patients’ characteristics.

Characteristics	Values
Age (years)	61 (46–77)
Male/female	3 (37.5) / 5 (62.5)
Weight (kg)	69 (57–79)
Smoking history	2 (25.0)
Tumor size (mm)	17 (10–38)
SUV max	7.0 (4.3–10.2)
TNR	2.9 (2.4–3.5)
Pathology	
Adenocarcinoma	8 (100.0)
Location	
Left lobe	2 (25.0)
Right robe	6 (75.0)
Pathological stage	
IA1	3 (37.5)
IA2	1 (12.5)
IA3	2 (25.0)
IB	2 (25.0)

Values are presented as median (range) or *n* (%).

TNR: tumor‐to‐normal tissue ratio.

Additionally, we also expanded our research scope to include pulmonary nodules in non‐lung cancer patients, such as pulmonary metastases from ovarian cancer, colorectal cancer, Ewing's sarcoma, and osteosarcoma (Figure S1, Supporting Information). The results showed that ICG can also specifically image these metastatic lesions, and most of them were taken up by macrophages, consistent with the results of primary lung cancer. This finding proved the similar role of macrophages in different tumor types, indicating that this may be a broad imaging mechanism, not just specific to a certain type of tumor.

### 2.4 Exploration of the Mechanism Underlying Macrophage‐Driven ICG Uptake

**Figure 4:**
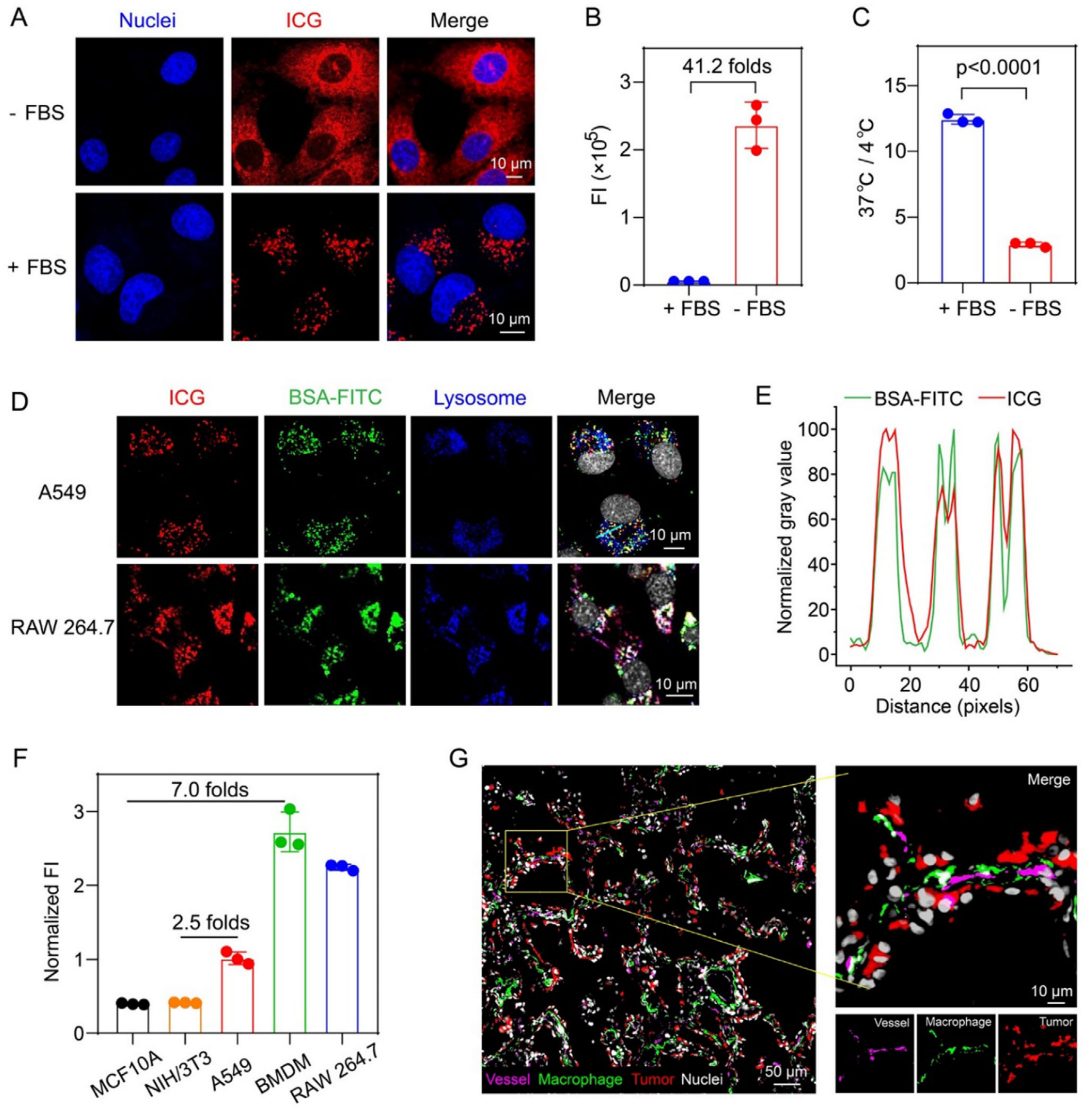
Investigation of ICG uptake mechanism. A) ICG uptake behavior in A549 cells under serum‐containing and serum‐free conditions. B) Flow cytometry quantification of ICG uptake in A549 cells under serum‐containing and serum‐free conditions. C) the ratio of ICG uptake in A549 cells at 37 °C compared to 4 °C. (*n* = 3 biologically independent cell samples, two‐sided Student *t*‐test). D) The uptake behavior and intracellular localization of ICG and BSA‐FITC. E) The extent of colocalization between ICG and BSA‐FITC along the cyan line in Figure 4D. F) The comparison of ICG uptake capacity in normal cells, tumor cells, and macrophages. (*n* = 3 biologically independent cell samples). G) The spatial distribution of tumor cells, macrophages, and blood vessels in lung cancer tissue. Nuclei, macrophages, tumor cells, and vessels were displayed in gray, green, red, and purple pseudo‐color, respectively. All data were presented as mean ± s.d.

Building on our above findings, we delved into the reasons behind the elevated ICG uptake in macrophages. It is well‐established that ICG rapidly binds to plasma proteins post‐injection into the human body, a fact that has been extensively researched and documented.^[^ ^19^ ^]^ Initially, we verified this phenomenon and investigated the detailed distribution patterns of ICG within cells. Specifically, we assessed ICG uptake under both serum‐rich and serum‐deprived conditions, observing notable variances in ICG distribution within A549 lung cancer cells. In the absence of serum, ICG exhibited a uniform distribution, whereas the presence of serum induced a punctate pattern (**Figure** **4**A). Moreover, in both A549 and bone marrow‐derived macrophages (BMDMs), ICG uptake in serum‐free conditions was dramatically higher—41 times and 70 times, respectively—compared to uptake in the presence of serum (Figure 4B; Figure S2A, Supporting Information). We then examined whether the uptake of ICG by A549 lung cancer cells was energy‐dependent. The results showed that in the presence of serum, the uptake of ICG at 37 °C was 12.4 times higher than that at 4 °C, while in the absence of serum, it was only 2.9 times higher (Figure 4C). This suggested that ICG uptake in the presence of serum was energy‐dependent, and macrophages exhibited a similar trend (Figure S2B, Supporting Information). From these observations, we indicated that under serum‐free conditions, ICG primarily entered cells via a non‐energy‐dependent free diffusion mechanism. In contrast, under serum conditions, ICG was internalized through endocytosis after binding to proteins, a process that requires energy. We then used FITC‐labeled BSA protein to further validate the uptake behavior of ICG under serum conditions. After co‐culturing cells with a mixture of ICG and BSA‐FITC, we noted a robust colocalization of ICG and BSA within lysosomes, confirming that ICG is taken up by tumor cells and macrophages through endocytosis after binding to BSA (Figure 4D,E). However, when examining the subcellular distribution of ICG under serum‐free conditions, we found the majority of it localized to the endoplasmic reticulum (Figure S3, Supporting Information), presenting a completely different intracellular distribution from that under serum conditions.

Having confirmed the uptake mechanism of ICG, we proceeded to assess its uptake capacity across normal cells, tumor cells, and macrophages. Our findings indicated that tumor cells exhibit an uptake capacity over 2.5 times that of normal cells, while macrophages demonstrate an even more pronounced capacity, being 7.0 times higher than that of normal cells. This suggests that the macrophages' elevated uptake capacity could be a significant factor in tumor imaging (Figure 4F). To explore whether the difference in uptake between macrophages and tumor cells was due to distinct endocytic mechanisms, we utilized a range of endocytic pathway inhibitors, including EAPI, chlorpromazine, nystatin, and dynasore. The findings indicated no significant differences in the endocytic mechanisms between the two cell types (Figure S4, Supporting Information). Additionally, to further investigate the interaction between ICG‐albumin complexes and macrophage‐specific receptors, we employed scavenger receptor (SR) inhibitors including polyinosinic acid, BLT‐1, and atorvastatin (Figure S5, Supporting Information). We found that BLT‐1 reduced macrophage uptake by ≈70%, suggesting that SR‐B1 may serve as a major pathway. Polyinosinic acid and atorvastatin showed milder reductions. These results collectively suggest that scavenger receptors, particularly those sensitive to BLT‐1, are likely involved and may play a significant role in mediating the uptake of ICG‐albumin complexes by macrophages.

Notably, our examination of the spatial interplay among tumor cells, macrophages, and blood vessels within lung cancer tissue revealed that macrophages are often situated adjacent to blood vessels, effectively forming a barrier between the vessels and the tumor cells (Figure 4G). Consequently, following intravenous injection, ICG rapidly binds to albumin, forming nano‐sized complexes. These complexes are then passively targeted to tissues via the EPR effect.^[^ ^11^ ^]^ Once the ICG‐albumin complexes extravasate from the blood vessels, they may first encounter macrophages and are largely intercepted by them, leaving minimal ICG to reach the tumor cells. This observation is also supported by studies such as that by Opzoomer et al., who identified a subset of tumor‐associated macrophages expressing lymphatic endothelial hyaluronan receptor 1 (Lyve‐1) as being spatially close to blood vessels.^[^ ^20^ ^]^ Other studies have also shown that stromal cells like macrophages are positioned around blood vessels, acting as a barrier for the delivery of nanodrugs to tumors.^[^ ^21^ ^]^ Therefore, the high ICG uptake by macrophages can be ascribed to both their inherent high uptake capacity and their strategic location relative to blood vessels.

### 2.5 Depletion of Macrophages in PDX Mice to Further Validate the Mechanism

**Figure 5:**
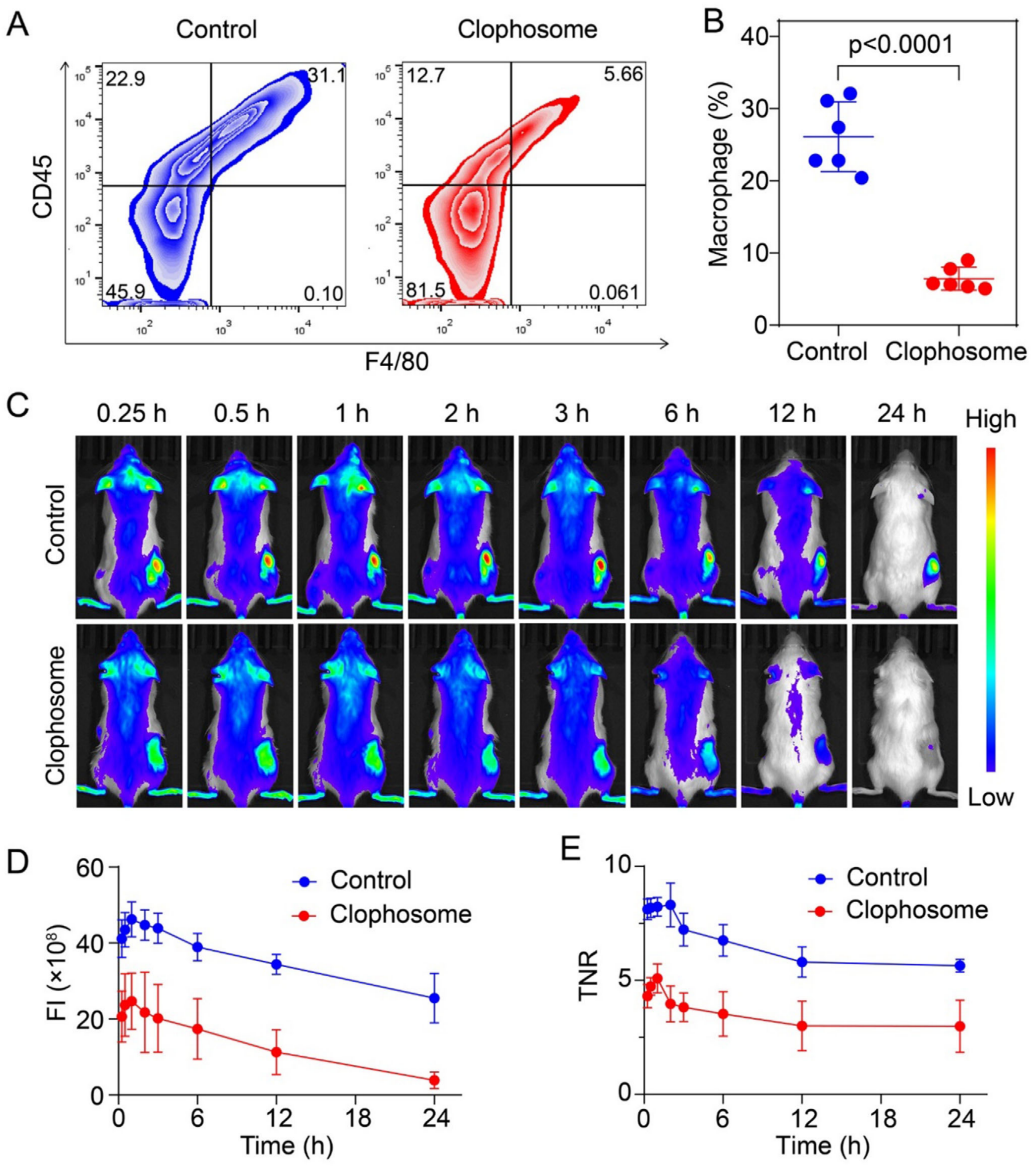
The in vivo ICG imaging of PDX tumor models with or without macrophage depletion. A,B) Flow cytometry (A) and quantitative results (B) for the macrophage content of tumor areas in the clophosome‐treated group and control group. (*n* = 6 mice per group, two‐sided Student *t*‐test). C) In vivo fluorescence images of the PDX tumor models at different time post‐injection of ICG. D) The quantitative ICG accumulation in tumors at 0.25, 0.5, 1, 2, 3, 6, 12, and 24 h post‐injection, respectively. E) The quantified fluorescence‐based Tumor‐to‐Normal Ratio (TNR) at 0.25, 0.5, 1, 2, 3, 6, 12, and 24 h post‐injection, respectively. All data were presented as mean ± s.d.

Subsequently, we developed lung cancer patient‐derived xenograft (PDX) mouse models and employed clophosome to selectively deplete macrophages, aiming to substantiate the macrophages' role in enhancing ICG imaging during surgery. After macrophage depletion with clophosome, we observed a significant reduction in macrophage levels at the tumor site in the treated group, which dropped to roughly one‐fourth of that in the control group, confirming the efficacy of our depletion strategy (**Figure** **5**A,B). Next, we administered ICG via tail vein injection to both the macrophage‐depleted and control PDX mice, capturing in vivo imaging at various time points post‐injection. The accumulation of ICG in the tumor region peaked within the first hour and then exhibiting a steady decline (Figure 5C). Importantly, the ICG accumulation in the depleted group was consistently and significantly lower than in the control group across all measured time points, highlighting the macrophages' critical role in ICG tumor imaging (Figure 5D). Furthermore, the tumor‐to‐normal ratio (TNR) was considerably lower in the macrophage‐depleted group when compared to the control group (Figure 5E). This is due to the tumor‐targeted macrophage depletion of clophosome. Liposomes can passively accumulate in tumors via the EPR effect, leading to higher local concentrations than in normal tissues. Importantly, our results showed a marked difference: macrophages in tumors were reduced by approximately fourfold after clophosome treatment (Figure 5A,B), whereas muscle tissue macrophages exhibited only a minimal reduction (<2‐fold) (Figure S6, Supporting Information). This sharp differential—highlighting both potent tumor microenvironment depletion and minimal off‐target effects in normal tissues—directly explains the decreased TNR. These findings not only demonstrate macrophages' essential role in imaging efficacy, but also indicate clophosome's potential for tumor‐selective depletion.

However, the accumulation and retention of ICG in tumor sites is a complex process, influenced by multiple factors. Besides the content of macrophages, the EPR effect also plays a certain role. Specifically, the EPR effect will likely influence the initial uptake, and the macrophage load may define the duration of retention. The macrophage's proximity to the vasculature facilitates the internalization of ICG that has extravasated through the EPR effect, thereby enhancing the overall accumulation of ICG in the tumor tissue. Therefore, the two are closely related and work synergistically. Consequently, we have also investigated the contributions of the EPR effect to ICG accumulation at various time points. The result showed that at 20 min post‐injection, the fluorescence intensity difference between washed and unwashed samples was 1.81‐fold, which decreased to 1.22‐fold by 24 h (Figure S7, Supporting Information). This indicated that during the early stages post‐ICG injection, the EPR effect makes a substantial contribution to the ICG signal detected at the tumor site. As the amount of unbound ICG in the extracellular space diminishes over time, at 24 h post‐injection, the ICG signal at the tumor site is predominantly derived from intracellular internalization. During this phase, the retention of ICG by macrophages plays a crucial role. The reason for this may be the very rapid plasma clearance of ICG, with a plasma half‐life of ≈3 min, and ≈97% being excreted from the blood 20 min post‐injection. Therefore, at later time points (24 h), the retention of ICG by macrophages in the tumor becomes the dominant factor.

### 2.6 Varying Macrophage Content Contributes to the Clinical ICG Imaging Differences

**Figure 6:**
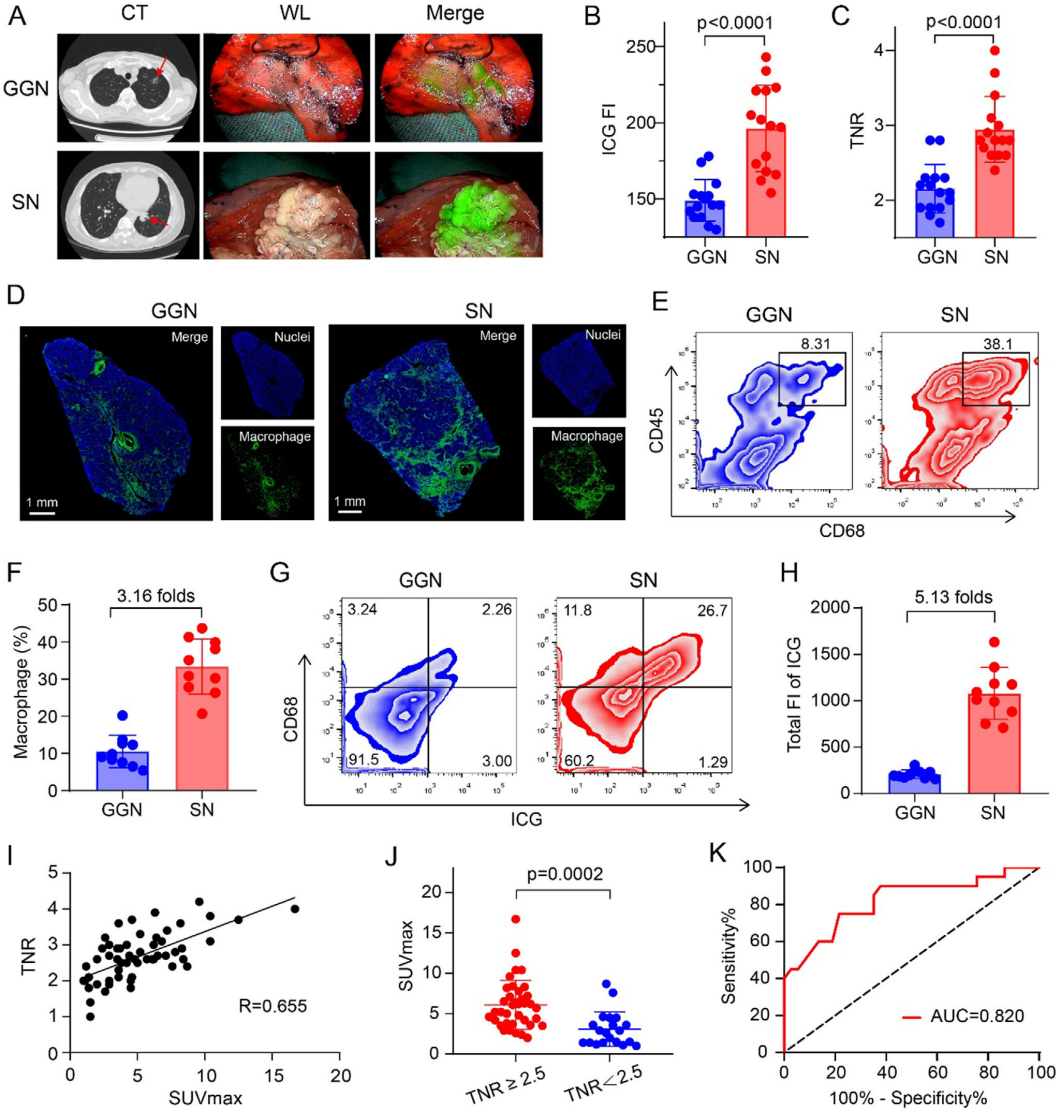
Variations in macrophage content contribute to the clinical ICG imaging differences for solid and ground‐glass pulmonary nodules. A) Computed tomography, visible light images, and fluorescence images of SN and GGN. B) Quantitative results of ICG fluorescence intensity at tumor sites (*n* = 15 patient/group). C) Quantitative results of ratio between tumor and normal tissue (TNR) (*n* = 15 patient/group). D,E) The immunofluorescence staining (E) and flow analysis (F) of macrophage content in patient samples. F) Quantitative macrophage percentages of GGNs and SNs, respectively (*n* = 10 patient/group). G) Flow cytometry assessment of ICG uptake in lung cancer samples. H) Quantitative results of total ICG uptake in lung cancer samples. Data were presented as mean ± s.d. I) TNR of ICG fluorescence imaging was positively correlated with SUVmax. (*n* = 57 patients). J) The comparison of SUVmax from lung cancer patients with TNR≥2.5 and TNR<2.5. K) ROC curves were plotted with sensitivity percentage versus 100%‐specificity percentage using an SUVmax threshold of 3.7, with patients having an ICG fluorescence imaging tumor to normal ratio below 2.5 considered as the negative group.

Clinically, the intraoperative ICG imaging outcomes are highly variable, with various patient and tumor characteristics affecting its success. In the context of intraoperative imaging for clinical lung cancer patients, we observed a notable disparity between the imaging effects of solid nodules (SNs) and ground‐glass nodules (GGNs). SNs demonstrated superior imaging characteristics, with ICG showing a marked concentration in the tumor region, creating a clear demarcation against the normal tissue and effectively highlighting the tumor's morphology (**Figure** **6**A). In contrast, GGNs exhibited poor ICG accumulation, resulting in a scattered distribution that obscured the tumor margins. Considering the differences in imaging characteristics, we randomly enrolled 15 patients with ground‐glass nodules and 15 patients with solid nodules, respectively. The quantitative analysis of intraoperative imaging data from 30 patients revealed that both the accumulation of ICG in tumor sites and the contrast ratio between tumor and normal tissue in solid nodules are significantly higher than those in GGNs (Figure 6B,C), aligning with our previous research conclusions.^[^ ^22^ ^]^

We subsequently investigated the cellular basis for this differential imaging efficacy. Immunofluorescence mapping revealed markedly increased macrophage infiltration in SNs versus GGNs (Figure 6D). Quantitative flow cytometric analysis corroborated these findings, demonstrating threefold greater macrophage density in SN specimens (Figure 6E,F). These data led us to hypothesize that the difference in macrophage content is a key factor contributing to the distinct ICG imaging effects observed between the two nodule types. To validate this hypothesis, we further investigated the uptake and distribution of ICG in tumors using flow cytometry experiments. The results demonstrated that the percentage of ICG‐positive cells in SNs is 28.0%, a substantial increase compared to the 5.26% observed in GGNs (Figure 6G). In addition, Macrophages were identified as the main group of ICG‐positive cells, evidenced by the significant presence of cells dual‐positive for both ICG and CD68. And the total intratumoral ICG content in SNs exceeded GGN levels by 5.13‐fold (Figure 6H). This finding strongly supports our hypothesis that macrophage‐dependent uptake mechanisms drive differential ICG accumulation, with a higher macrophage content corresponding to an increased population of cells with high ICG uptake, thereby significantly enhancing the accumulation of ICG in tumor regions.

Further delineating the macrophage heterogeneity, we investigated the contribution of M1 and M2 polarized subtypes to ICG uptake. Immunofluorescence co‐staining revealed that intracellular ICG signals predominantly colocalized with M2 macrophages, while exhibiting minimal overlap with M1 macrophages, across both GGN and SN samples (Figure S8A, Supporting Information). This subtype‐specific association was corroborated by flow cytometric quantification (Figure S8B–D, Supporting Information). Notably, M2 macrophages constituted the major subpopulation, representing 23.9% and 47.4% of the total CD68⁺ macrophages in GGNs and SNs, respectively. In contrast, M1 macrophages accounted for only 3.90% (GGNs) and 1.62% (SNs). Consequently, the M2/M1 ratio was significantly higher in SNs compared to GGNs (Figure S8D, Supporting Information). These findings suggest that the enhanced ICG accumulation observed, particularly in SNs, is likely driven, at least in part, by selective uptake within the M2‐polarized macrophage subset. This observed M2 prevalence could provide a cellular mechanism contributing to the macrophage‐dependent differential ICG retention between GGNs and SNs.

The experiments mentioned above have verified that the macrophage content may serve as a biomarker to predict the intraoperative ICG imaging effect and has the potential for clinical translation. These findings not only provide a cellular basis for understanding the differential imaging efficacy but also open up new possibilities for preoperative prediction and patient selection in clinical practice. Building on the work of Reinfeld et al., who have reported a correlation between the maximum standardized uptake value (SUVmax) and macrophages.^[^ ^23^ ^]^ A retrospective analysis was conducted, including 57 patients from Peking University People's Hospital who underwent preoperative PET‐CT and near‐infrared fluorescence imaging between March 2022 and October 2024. Linear regression analysis demonstrated a positive correlation between the tumor‐to‐normal ratio (TNR) and SUVmax, suggesting that it may be feasible to predict the intraoperative ICG imaging outcomes for patients based on their preoperative PET/CT scan data (Figure 6I).

Additionally, we further speculated a SUVmax cut‐off of 3.7 that may reliably predict the surgical utility of ICG. In various studies on fluorescence‐guided surgery, different centers adopt distinct Tumor‐to‐normal ratio (TNR) thresholds to define the success of fluorescence imaging. Previous reports suggested that an TNR > 2 is generally sufficient for the clear visualization of fluorescent targets. During thoracoscopic surgery for pulmonary nodules, tumor depth significantly impacts imaging effectiveness. In clinical practice, higher TNR are required to achieve successful fluorescence‐guided surgery in the thoracic surgery. Based on previous research and the surgical expertise of our center, this study established a TNR greater than 2.5 as the threshold for successful intraoperative imaging. Our statistical analysis revealed a significant difference in SUV values when comparing TNRs above and below the 2.5 threshold (Figure 6J). Using a TNR of 2.5 as the threshold for successful intraoperative imaging with ICG, the corresponding SUV cut‐off value was 3.7, and the ROC curve analysis results show that prediction based on this SUV cut‐off has high accuracy (AUC = 0.820) (Figure 6K). Given that other factors such as tumor size and histologic subtypes may influence the relationship between SUVmax and TNR, we performed multivariable linear regression analyses to adjust for these potential confounders. The results showed that even after adjustment, SUVmax remained significantly and independently associated with TNR (Table S1, Supporting Information). Additionally, CT imaging characteristics (SN vs GGN) and histologic subtypes (squamous cell carcinoma vs adenocarcinoma) also showed a significant association with TNR. These findings can assist surgeons in preoperative patient selection, enabling the identification of more suitable candidates for thoracoscopic fluorescence‐guided lung cancer surgery. However, considering the application scope, this technique may be restricted in clinics or hospitals lacking advanced PET‐CT scanners. Thus, it is crucial to develop a more accessible method for selecting patients for ICG‐guided surgery. Histopathological evaluation of macrophage content through post‐biopsy immunohistochemical analysis provides a practical alternative for clinical decision‐making.

## 3 Discussion

Despite the benefits of ICG fluorescence navigation in lung cancer surgery, its imaging mechanism and optimal application are still not fully understood, with various patient and tumor characteristics affecting its success.^[^ ^24^ ^]^ This study deeply explores the fluorescence imaging mechanism of ICG in lung cancer surgery and reveals an important phenomenon: macrophages are the main reservoirs of ICG, rather than tumor cells. This finding not only provides us with a new perspective to understand the imaging mechanism of ICG, but also provides a theoretical basis for optimizing the application of ICG in lung cancer surgery. In our study, we found that up to two‐thirds of the total amount of ICG in lung cancer tissues is taken up by macrophages, while the uptake of ICG by tumor cells is less than 10%. More specifically, when we focus on the ICG uptake level of individual cells, the uptake of macrophages is about seven times that of tumor cells. This significant difference highlights the central role of macrophages in high‐contrast imaging of ICG during lung cancer surgery. It also challenges our current understanding of the imaging mechanism of ICG, showing that the main distribution of ICG in lung cancer tissues was not limited to tumor cells as expected by traditional concepts,^[^ ^11^, ^16^ ^]^ but rather had a higher concentration in macrophages. Although some studies have also explored the interaction between ICG and macrophages, the focus was primarily on the field of atherosclerosis. These studies showed that ICG can bind to albumin or lipoproteins to target macrophages in atherosclerotic plaque regions, enabling early diagnosis of atherosclerosis and monitoring of treatment efficacy.^[^ ^17^ ^]^ However, for cancer intraoperative fluorescence imaging, there is a notable absence of research that systematically examines the detailed distribution of ICG among different cell types within tumor tissues and its interactions with these various cellular components. Our research has addressed the limitations of previous studies by systematically examining the distribution of ICG within tumor tissues, revealing the crucial role of macrophages in ICG enrichment.

We also delved into understanding why macrophages become the primary reservoir for ICG in lung cancer tissue. The results showed that the heightened uptake in macrophages and the advantageous geographical position of macrophages adjacent to tumor blood vessels are two major reasons. Additionally, the macrophage depletion experiments conducted on PDX mouse models derived from lung cancer patients further underscores the crucial role of macrophages in achieving high‐contrast ICG imaging of tumors. Numerous studies have reported a higher presence of macrophages in tumor tissues compared to normal tissues,^[^ ^25^ ^]^ and our experiments have confirmed this as well. This elevated macrophage density also contributes to the enhanced tumor imaging contrast provided by ICG. Furthermore, we noted that in clinical lung cancer patients, solid nodules exhibit better ICG imaging than ground‐glass nodules. This finding aligns with our previous research and observations by other researchers,^[^ ^12^, ^14^, ^22^ ^]^ though the precise mechanism behind this imaging difference had not been clearly elucidated before. In this study, we discovered a pronounced abundance of macrophages in SNs (SN 38.1% vs GGN 8.31%) and identified that the disparity in macrophage content is a pivotal factor contributing to the clinical differences in ICG imaging between SNs and GGNs. Notably, we observed a positive correlation between the fluorescence‐based TNR and SUVmax derived from ^18^F‐FDG‐PET/CT scans, suggesting a possibility of forecasting the success of ICG imaging through preoperative PET/CT scans. Additionally, we further speculated a SUVmax cut‐off of 3.7 that may reliably predict the surgical utility of ICG. This value may provide a reference for surgeons to select patients who are suitable for thoracoscopic fluorescence‐guided lung cancer surgery prior to the operation.

However, there are still some limitations in this study. First, the accumulation and retention of ICG at the tumor site is a complex process influenced by various factors. EPR effect likely influence the initial accumulation of ICG, and the macrophage content may define the duration of retention. Although the rapid plasma clearance of ICG means that the contribution of EPR is relatively low when imaging 24 h post‐administration, it must be acknowledged that this contribution was somewhat overlooked in this study. Instead, our study primarily focused on the retention of ICG in tumor tissues, including the main cell types involved in its uptake, and the underlying mechanisms. Second, this study mainly focuses on macrophages and tumor cells, with limited discussion on the potential impact of other cell types on ICG imaging, such as fibroblasts, endothelial cells, and various immune cells. Subsequent research should expand to these cell types to fully reveal the imaging mechanism of ICG in lung cancer surgery, thereby providing a deeper understanding for clinical applications. Third, the SUVmax cut‐off value of 3.7 identified in this single‐center study requires external validation to confirm its generalizability and robustness. Factors such as variations in imaging protocols, scanner calibration, and patient populations across different institutions could potentially impact the optimal threshold. Therefore, we propose that future work should include a prospective, multicenter validation study. Such a study, involving diverse clinical settings with standardized imaging and analysis protocols, is essential to establish the reliability and broader applicability of this threshold for guiding ICG‐based tumor identification in lung cancer surgery.

In summary, this study delves into the mechanism of ICG in lung cancer surgery imaging, revealing that macrophages, rather than tumor cells, serve as the main reservoir for ICG. Based on this finding, we provided a deeper understanding of the mechanisms behind the superior imaging efficacy of solid nodules over ground‐glass nodules, and proposed the possibility of predicting the intraoperative ICG imaging outcomes through preoperative PET scans or by measuring macrophage content via needle biopsy. This discovery not only provides novel avenues for optimizing the application of ICG in surgical procedures but also offers valuable insights into the exploration of personalized treatment methods.

## 4 Experimental Section

### Materials

ICG was supplied by Yichuang Pharmaceutical Limited Liability Company (Dandong, China). Clophosome® was purchased from Dakewe Biotech Co., Ltd. Hoechst 33342, LysoTracker®Red DND‐99, ER‐Tracker Green were obtained from Invitrogen. PE anti‐human CD45 (304008) antibody, AF647 anti‐human EpCAM (369820) antibody, PE/Cyanine7 anti‐human CD86 antibody (374210), and Brilliant Violet 421™ anti‐human CD206 antibody (321126) were purchased from Biolegend. Anti‐CD68 (ab303565), Anti‐CD163 (ab182422), anti‐EpCAM (ab187372 and ab282457), anti‐F4/80 (ab16911), anti‐CD31 antibody (ab9498), FITC anti‐F4/80 antibody (ab60343), AF488‐conjugated Goat anti‐Rat (ab150157), and AF594‐conjugated Goat anti‐Mouse (ab96873) secondary antibodies were all purchased from Abcam. Other solvents and reagents were received from Sigma‐Aldrich or Fisher Scientific Inc.

### Cell Lines

The A549 lung carcinoma (1101HUM‐PUMC000002), RAW264.7 macrophage (3101MOUSCSP5036) were obtained from the National Infrastructure of Cell Line Resource. A549 cells were cultured in DMEM supplemented with 10% FBS and antibiotics under 5% CO_2_ atmosphere at 37 °C. RAW 264.7 cells were cultured with a heat‐inactivated DMEM medium. In addition, murine bone marrow‐derived macrophages (BMDMs) were isolated from surgically excised femurs and tibias of female C57BL/6 mice (aged 6–8 weeks). These BMDMs were then cultured in a complete heat‐inactivated DMEM medium supplemented with recombinant murine macrophage colony‐stimulating factor (m‐CSF, 20 ng mL^−1^, PeproTech) for a duration of 5 days.

### Cellular ICG Uptake

A confocal laser scanning microscope (CLSM, Leica STELLARIS 8) and flow cytometry (FCM, Beckman CytoFLEX LX) were utilized to assess the cellular uptake of ICG in tumor cells (A549) and macrophages (RAW 264.7 and BMDM). Initially, the cells were seeded in glass‐bottom dishes and incubated overnight to allow for attachment. Subsequently, the cells were rinsed with PBS and incubated in either incomplete medium (devoid of FBS) or complete medium (containing 10% FBS), both supplemented with ICG (1 µg mL^−1^). Following an incubation period of 1 h at 37 °C, the cells were washed three times with PBS buffer and stained with Hoechst 33342 for analysis using the CLSM. The collected parameters of ICG channel were as follows: excitation wavelength at 780 nm, emission wavelength window of 795–850 nm, laser intensity set to 20 (scale of 0–100), and gain value set to 100 (scale of 0–1000). For quantitative analysis of cellular uptake through FCM, cells were seeded in 12‐well plates at a density of 2 × 10^5^ cells per well and incubated overnight. Afterward, the cells were incubated with ICG in either incomplete or complete medium. Subsequently, the cells were washed three times with cold PBS and subjected to FCM for quantification. The parameters were set as follows: excitation wavelength at 808 nm, with an 840/20 BP filter, and a gain value set to 100 (scale of 1–3000).

### Subcellular Trafficking of ICG

Cells were plated in glass‐bottom dishes and incubated in incomplete medium supplemented with ICG (1 µg mL^−1^) and BSA‐FITC (1 mg mL^−1^) for 1 h. Following this incubation, the cells were thoroughly washed three times with PBS buffer. Subsequently, Hoechst 33342 and Lyso tracker Red were added to label the cell nuclei and lysosomes, respectively. After an additional 15‐min incubation, the images were captured using a confocal laser scanning microscope (CLSM).

### Patient‑Derived Xenograft (PDX) Mice Model Establishment

The fresh tumor specimens were obtained from lung cancer patients who had undergone surgical treatment at Peking University People's Hospital. Subsequently, these tumor samples were thoroughly cleansed by immersion in a phosphate‐buffered saline (PBS) solution. These samples were then dissected into small cubes and implanted into the subcutaneous tissue of immunodeficient, specific pathogen‐free (SPF) mice (NOD‐Prkdc^null^‐Il2rg^null^) within 48 h to establish the PDX mice model. The tumor volume was monitored daily, and the experiment commenced once the tumor volume reached 150 mm^3^.

### Depletion of Macrophages In Vivo

To elucidate the impact of macrophages on ICG‐guided intraoperative fluorescence imaging, tumor‐associated macrophages were depleted by administering Clophosome® (FormuMax) intravenously. 0.2 mL of Clophosome® was injected per mouse via the tail vein 2 days prior to tumor inoculation (−2 days). On day 0, the tumor was inoculated. Thereafter, administration of 0.1 mL of clodronate liposomes per mouse was continued every 4 days until the tumor model was fully established, indicated by a tumor volume of 150 mm^3^. To ensure that macrophages were effectively cleared from the mice, an additional 0.1 mL dose of Clophosome® was administered 48 h before the in vivo imaging study. ICG was then intravenously injected at a dosage of 5 mg kg^−1^, and fluorescence images were captured at intervals of 15 min, 0.5, 1, 2, 3, 6, 12, and 24 h post‐injection using the IVIS Spectrum imaging system (PerkinElmer). Concurrently, the depletion of F4/80^+^ macrophages was validated through flow cytometry analysis.

### Clinical Imaging and Samples Collection

The study population comprised lung cancer patients who underwent surgical resection at the Peking University People's Hospital. All enrolled patients received an intravenous injection of ICG at a dose of 5 mg kg^−1^ body weight 24 h prior to the scheduled operation time and subsequently underwent lobectomy or sublobectomy as planned. The ICG administration timing and dosage protocol used in this study was based on previous research findings and the center's clinical experience. Before infusion, ICG was dissolved in 250 mL of saline and administered intravenously under light‐protected conditions, with the infusion completed within 30 min. The DPM‐III‐01 NIR thoracoscope system (Zhuhai Dipu Medical Technology Co., Ltd., China) was utilized for real‐time conventional and fluorescent imaging during surgery (Video S1, Supporting Information). Surgeons first identified suspicious pulmonary nodules by integrating preoperative computed tomography (CT) data combined with the conventional white‐light thoracoscopy. Subsequently, the nodules were evaluated using near‐infrared imaging to ascertain their fluorescent characteristics. All identified lesions were then thoroughly excised. The specific surgical method was determined according to the American Joint Committee on Cancer (AJCC) lung cancer treatment guidelines, including lobectomy and sublobectomy. To elucidate the mechanism underlying the specific intraoperative imaging of lung cancer tissues using ICG and to determine the type of cellular interaction responsible for the high accumulation of ICG at tumor sites, eight patients with superior imaging contrast were selected for this study. The resected fresh tumor specimens were collected and embedded in optimal cutting temperature compound (OCT), frozen using liquid nitrogen, and cut into 10 µm slice. The obtained tumor slice was then used for fluorescence immunofluorescence staining, H&E staining, and immunohistochemistry. To study the clinical differences in ICG imaging between solid nodules (SNs) and ground‐glass nodules (GGNs), a total of 30 patients were enrolled, with 15 each for SNs and GGNs. The ICG fluorescence at the tumor sites was qualified and the ratio of fluorescence intensity between the tumors and the surrounding normal lung tissue (TNR) was calculated. Additionally, a retrospective analysis of the correlation between the tumor‐to‐normal ratio (TNR) and SUVmax was conducted, including 57 patients from Peking University People's Hospital who underwent preoperative ^18^F‐FDG PET‐CT and near‐infrared fluorescence imaging between March 2022 and October 2024.

### ^18^F‐FDG PET/CT Imaging

Patients included in this study underwent whole‐body PET‐CT before surgery, aiding surgeons in assessing the pulmonary nodules and the overall systemic condition. Patients received an intravenous injection of 4.5–5.5 MBq/kg of ^18^F‐fluorodeoxyglucose (^18^F‐FDG). PET/CT scans were conducted using a GE Discovery VCT positron emission tomography/computed tomography (PET/CT) scanner, capturing whole‐body images ≈60 min post‐injection of the radiotracer.

### Immunofluorescence Staining

The tumor slices were first fixed in a 4% paraformaldehyde solution to ensure fixation and preservation of cellular structures. This was followed by a permeabilization step using a 0.1% Triton X‐100 solution for 5 min, allowing for better penetration of antibodies into the tissue. Subsequently, the slices were treated with a quick blocking solution for 1 h at room temperature to minimize non‐specific binding. Afterward, the tumor slices were subjected to immunofluorescence staining. They were incubated with a combination of primary antibodies: a mouse anti‐EpCAM antibody and a rat anti‐F4/80 antibody. This incubation was carried out at 4 °C overnight to allow for optimal antibody binding. On the subsequent day, the slices were further processed by adding secondary antibodies: AF488‐conjugated Goat anti‐Rat and AF594‐conjugated Goat anti‐Mouse antibodies. This staining step was performed at 37 °C for 2 h to ensure thorough binding of the fluorescently labeled secondary antibodies to their target antigens. Finally, the slides were stained with Hoechst 33342 and scanned by Leica confocal. The immunofluorescence staining of M1 and M2 macrophages was performed using the same method as described above.

### Immunohistochemistry

The patient‑derived tumor slices were fixed in 4% formaldehyde and permeabilized with 0.1% Triton X‐100. The tissue sections were then treated with a 3% hydrogen peroxide (H_2_O_2_) solution for 10 min at room temperature. This step effectively blocked peroxidase activity, ensuring a clean background for subsequent immunostaining. Subsequently, tissue sections were washed with PBS to remove any residual hydrogen peroxide and then stained with primary antibody. Next, sections were incubated with corresponding Poly‐HRP conjugated secondary antibodies at room temperature for 30 min. The visualization of antibody binding was achieved using the EnVision™ FLEX Working Solution (Dako Denmark A/S, Glostrup, Denmark), this reagent was applied to the sections for 10 min at room temperature, enabling the detection of the HRP‐labeled antibody‐antigen complexes. Then, the tumor slices were briefly counterstained with hematoxylin for 5 s, giving the cell nuclei a clear blue color that helped in examining the tissue's structure. Finally, the tumor slices were dehydrated using a graded ethanol series, and then coverslipped for microscopic examination.

### Flow Cytometry Analysis of Cell Population in Tumors

The fresh tumor tissues collected from lung cancer patient were harvested and subjected to enzymatic digestion using a solution containing 0.5 mg mL^−1^ collagenase I and 0.2 mg mL^−1^ DNAase I (Invitrogen, USA) for 30 min, and then ground by the rubber end of a syringe to facilitate the dissociation of the tissue. Then, the resulting cell mixture was filtered through nylon mesh sieves to create a homogeneous suspension and washed with PBS. To minimize nonspecific binding to Fc receptors (FcRs), which could interfere with subsequent antibody interactions, the single cell suspension was incubated with anti‐CD16/32 (Biolegend) for 15 min. The single cell suspension was then labeled with a combination of corresponding fluorescently conjugated antibodies. Finally, those cells were washed with PBS for two times to remove any unbound antibodies and then analyzed using flow cytometry.

## Conflict of Interest

The authors declare no conflict of interest.

## Ethics Statement

This research strictly complies with all relevant ethical standards. The studies involving patients have been approved by the Institutional Review Board of Peking University People's Hospital (approval no. 2021PHD012‐001), with all patients providing written informed consent. All animal procedures were performed according to the Guidelines approved by the Institutional Animal Care and Use Committee (IACUC) of Peking University (Accreditation number: LA2019039).

## Statistical Analysis

All experiments were performed in triplicate at least. Data were presented as mean ± standard deviation (s.d.) and analyzed using GraphPad Prism 8 and Origin 2022 software. Statistical significance among different groups was determined using Student's *t*‐tests for pairwise comparisons or one‐way (or two‐way) analysis of variance (ANOVA) followed by Tukey's post hoc test for multiple comparisons. For groups with unequal standard deviations, Welch's correction was implemented. Simple linear regression was used to analyze the relationship between clinicopathological characteristics and TNR. Variables with *p*‐values less than 0.1 in the univariate analyses were included in the multiple linear regression model. In all cases, statistical significance was set as follows: *p* < 0.05 was considered significant, *p* < 0.01 was regarded as highly significant.

## Author Contributions

Y.Y. and J.M. are co‐first authors and contributed equally to this work. Y.Y. and J.M. performed the experiments and wrote the manuscript; Y.L. and G.J. provided helpful discussions and methodology; Y.Z. assisted on cell culture and animal work; Y.L., H.Z., J.L., and X.Y. assisted with data analysis; K.C. and F.Y. helped with experimental design; J.W., Y.W., and J.Z. conceived the study and revised the manuscript. All authors reviewed, commented on, and approved the final version of this manuscript.

## Supporting information



Supporting Information

Supplemental Video 1

## Data Availability

The source data underlying Figures 2, 3, 4, 5, and 6 are provided with this paper. The main data that support the findings of this study are available within the paper, source data and its supplementary information. Other raw and relevant data during the study are available for research purposes from the corresponding authors upon reasonable request.
