# Supplementary material for: Unveiling Macrophage Content as a Predictive Biomarker for Intraoperative ICG Imaging Efficacy in Lung Cancer
Source: Adv Sci (Weinh). 2025 Aug 4;12(39):e04498. doi: 10.1002/advs.202504498 (PMC12533320; doi:10.1002/advs.202504498)

**Supplemental information**

**Unveiling Macrophage Content as a Predictive Biomarker for Intraoperative ICG Imaging Efficacy in Lung Cancer**

Yue Yan^1^^†^, Jiahui Mi^2,3†^, Yun Li^2,3^, Guanchao Jiang^2,3^, Yimeng Zhang^4^, Yanguo Liu^2,3^, Hui Zhao^2,3^, Jianfeng Li^2,3,5^, Xing Yang^6^, Jun Wang^2,3^, Fan Yang^2,3^*, Kezhong Chen^2,3^*, Yiguang Wang^4,7^*, Jian Zhou^2,3^*

Y. Yan^†^

Department of Central Laboratory, Peking University First Hospital; Beijing 100034, China

J. Mi^†^, Y. Li, G. Jiang, Y. Liu, H. Zhao, J. Li, J. Wang, F. Yang, K. Chen, J. Zhou

Department of Thoracic Surgery, Peking University People's Hospital; Beijing 100044, China

Email: yangfan@pkuph.edu.cn; chenkezhong@pkuph.edu.cn; [zhoujian@bjmu.edu.cn](mailto:zhoujian@bjmu.edu.cn)

J. Mi^†^, Y. Li, G. Jiang, Y. Liu, H. Zhao, J. Li, J. Wang, F. Yang, K. Chen, J. Zhou

Thoracic Oncology Institute, Peking University People's Hospital; Beijing, 100044, China

Y. Zhang, Y. Wang

State Key Laboratory of Natural and Biomimetic Drugs, Peking University; Beijing 100191, China.

Email: [yiguang.wang@pku.edu.cn](mailto:yiguang.wang@pku.edu.cn)

J. Li

Department of Thoracic Surgery, Peking University People's Hospital; Qingdao, 266111, China

X. Yang

Department of Nuclear Medicine, Peking University People's Hospital; Beijing 100044, China

Y. Wang

Beijing Key Laboratory of Molecular Pharmaceutics and New Drug Delivery System, School of Pharmaceutical Sciences, Peking University; Beijing, 100191, China.

This PDF file includes:

Figure S1 to S8, Table S1


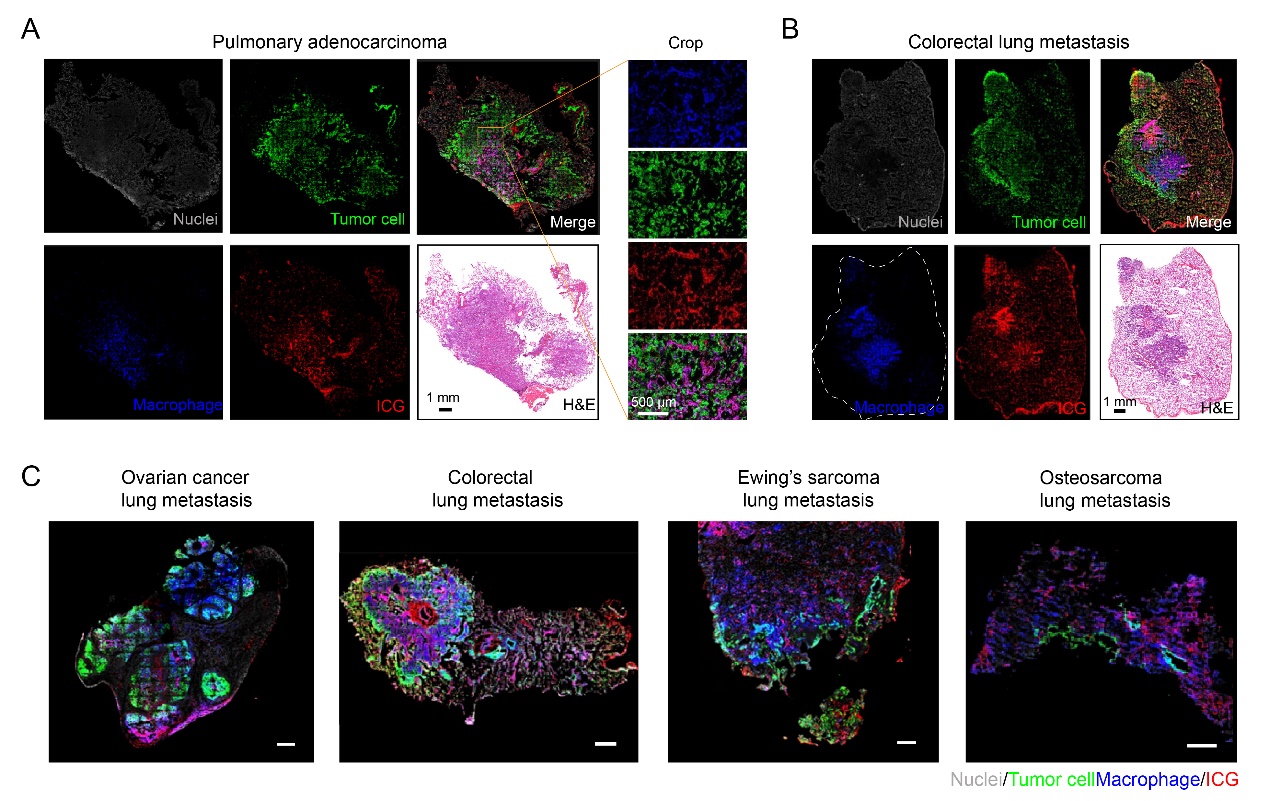


Figure S1. Dissecting the contribution of macrophage and tumor cells on ICG imaging. (A and B) Representative Immunofluorescence staining of pulmonary adenocarcinoma (A) and colorectal lung metastasis patient (B), respectively. H&E staining was utilized to confirm the tumor's location. (C) Investigation of ICG co-localization with tumor cells and macrophages in non-lung cancer patients, including pulmonary metastases from ovarian cancer, colorectal cancer, Ewing’s sarcoma, and osteosarcoma. The nuclei, macrophages, tumor cells, and ICG were displayed in gray, blue, green, and red pseudo-color, respectively. Scale bar = 1 mm.


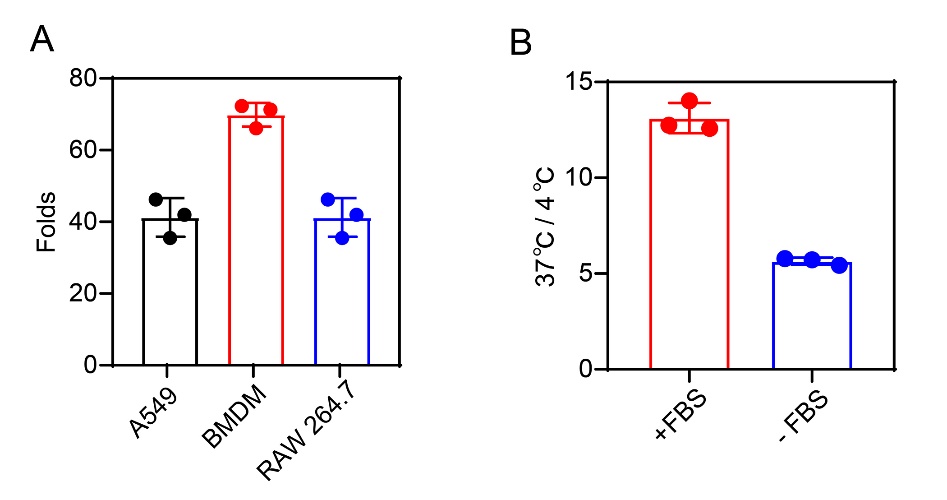


Figure S2. Investigation of ICG uptake mechanism. (A) Flow cytometry quantification of ICG uptake folds in serum-free conditions compared to uptake under serum conditions. (n = 3 biologically independent cell samples) (B) The ratio of ICG uptake in RAW 264.7 cells at 37 ℃ compared to 4 ℃. (n = 3 biologically independent cell samples). All data were presented as mean ± s.d.


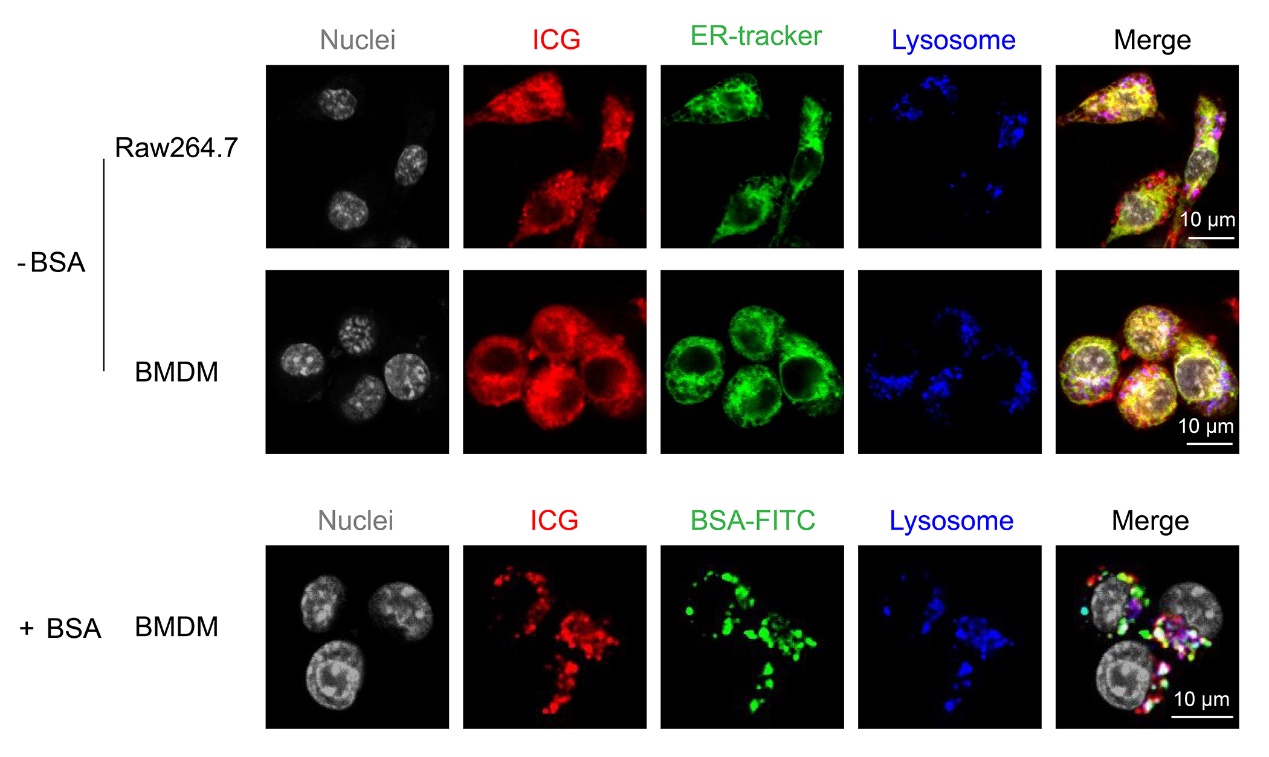


Figure S3. ICG uptake behavior under BSA-containing and BSA-free conditions.

.


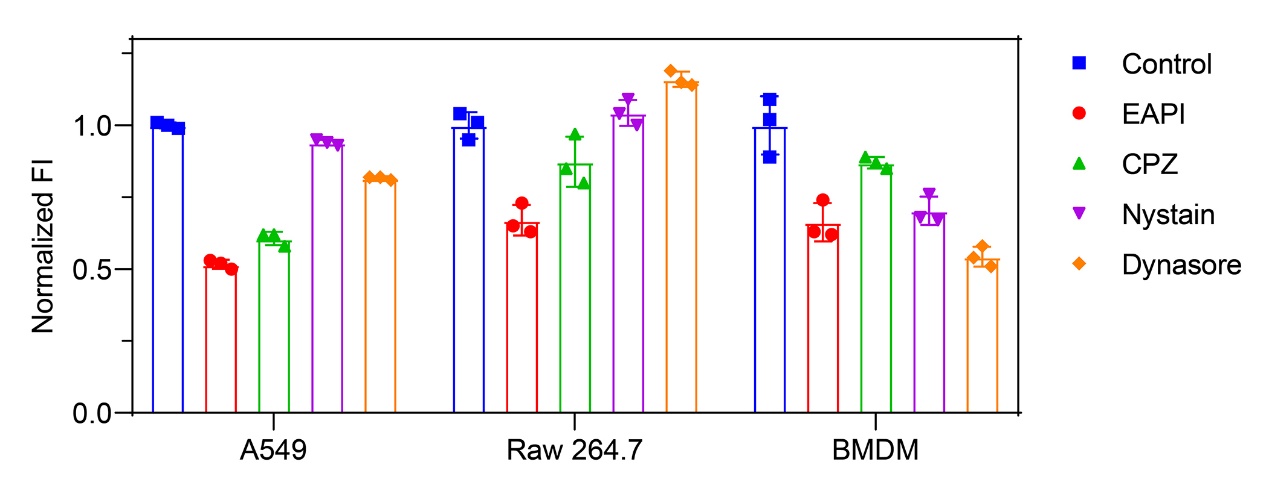


Figure S4. The endocytic mechanisms investigation of tumor cells and macrophages, employing endocytic pathway inhibitors such as EAPI, chlorpromazine, nystatin, and dynasore. (n = 3 biologically independent cell samples). Data were presented as mean ± s.d.


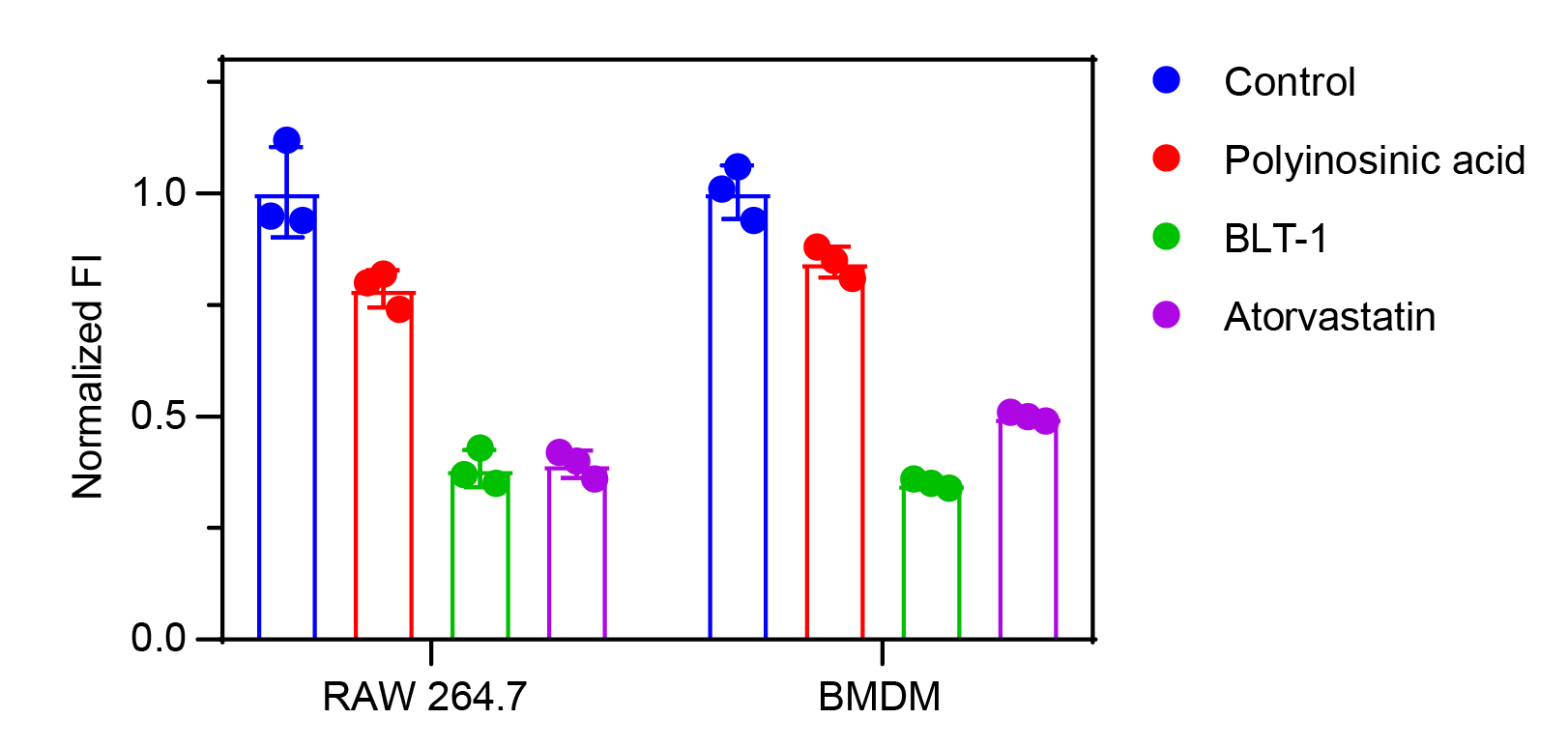


Figure S5. The investigation of the interaction between ICG-albumin complexes and macrophage-specific receptors, employing scavenger receptor inhibitors including polyinosinic acid, BLT-1, and atorvastatin (n = 3 biologically independent cell samples). Data were presented as mean ± s.d.


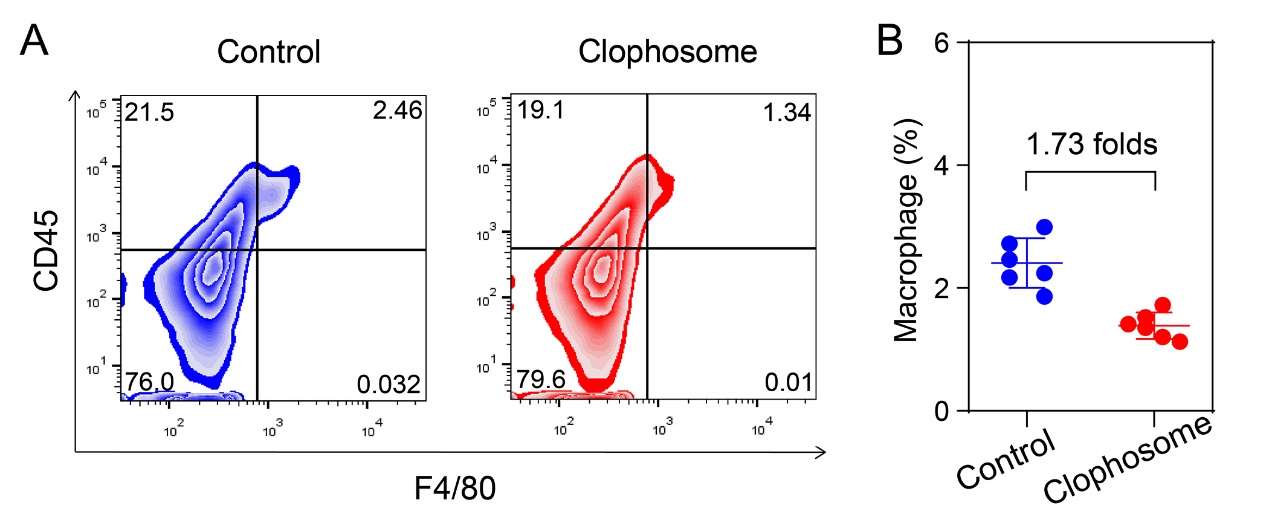


Figure S6. Flow cytometry (A) and quantitative results (B) for the macrophage content of normal tissue (muscle) in the clophosome-treated group and control group of lung cancer patient-derived xenograft (PDX) mouse models. (n = 6 mice per group). Data were presented as mean ± s.d.


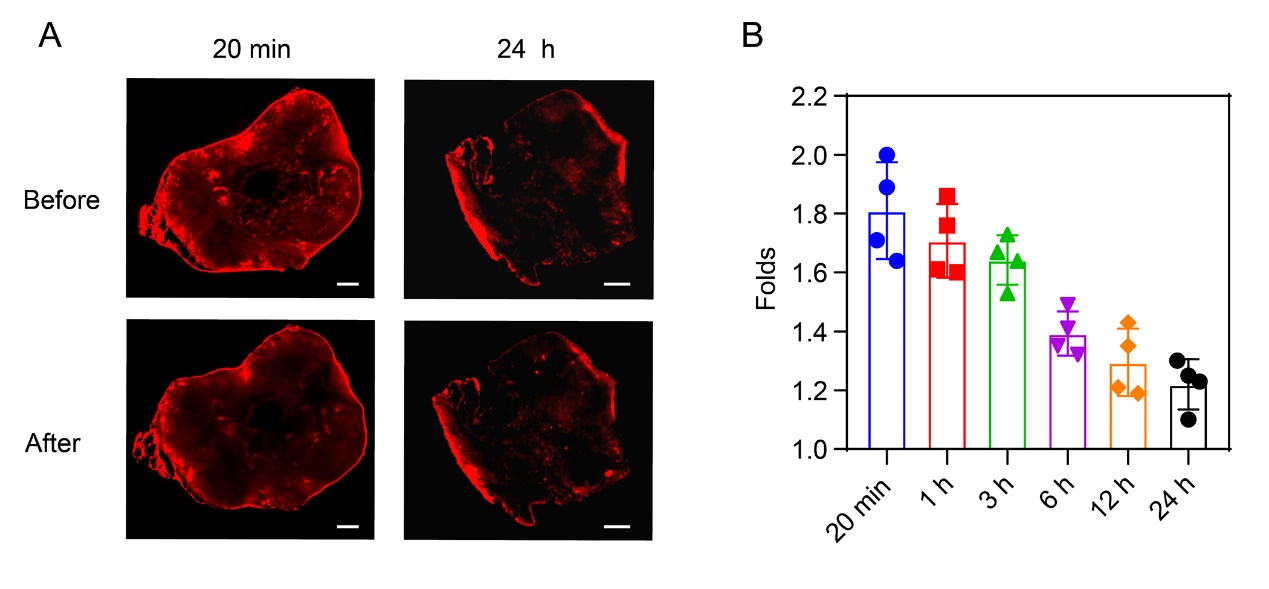


Figure S7. The comparison of ICG fluorescence in PBS washed and unwashed tumor slice. Scale bar=500 μm. (A) Representative images at 20 min and 24 h after ICG injection, both using PBS washing and no washing methods, respectively. (B) ICG fluorescence intensity ratios of unwashed tumor slices to washed tumor slices at different time points after ICG injection. Data were presented as mean ± s.d. (n = 4 mice per group)


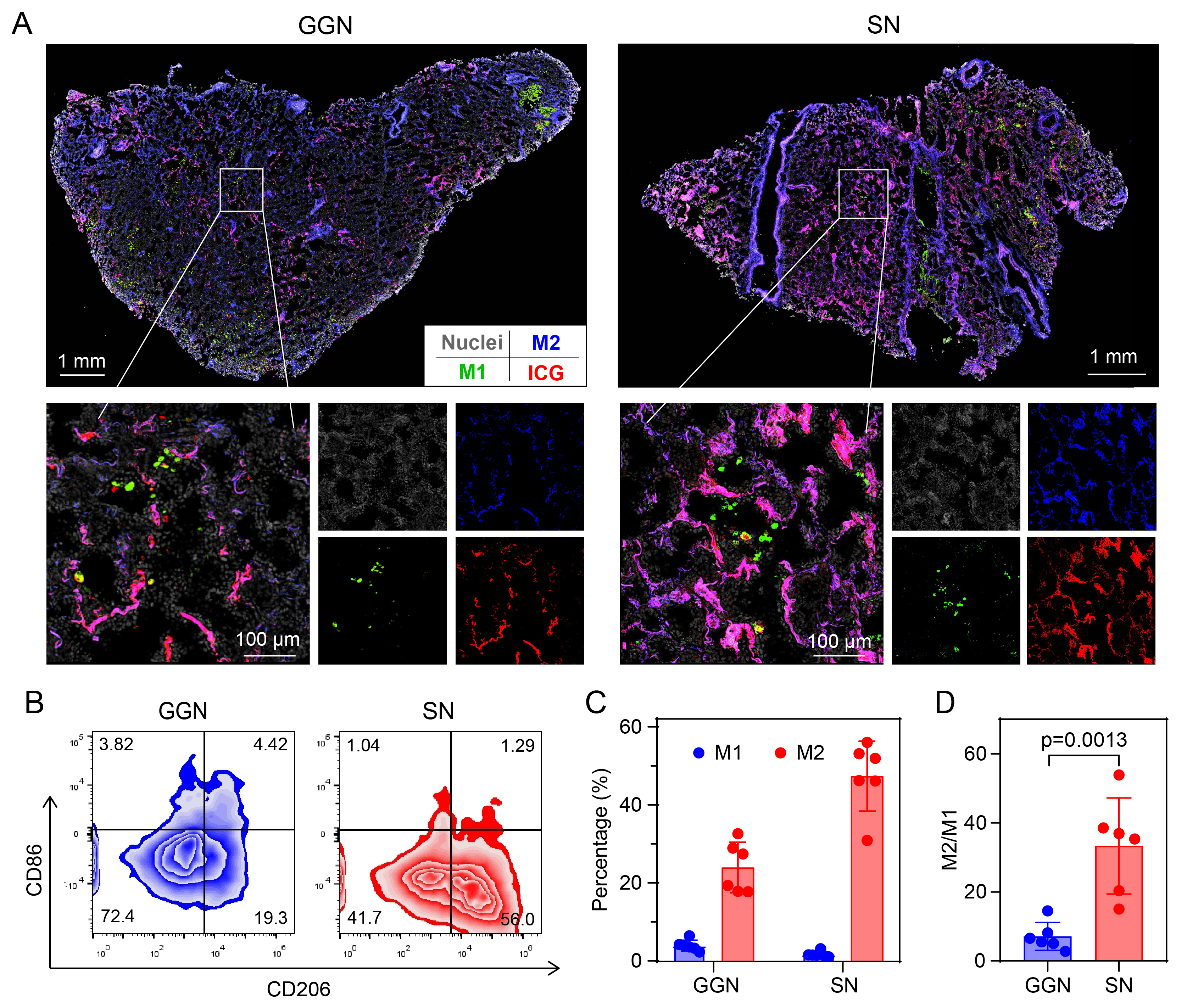


Figure S8. The co-localization between ICG and M1/M2 macrophages. (A) Full-slide scanning of the lung cancer tissue sections that were immunofluorescently stained with M1 and M2 type macrophage cells. The nuclei, M1 macrophages, M2 macrophages, and ICG were displayed in gray, green, blue, and red pseudo-color, respectively. (B) **The flow analysis of M1 and M2 macrophage content in patient samples (gated on CD45⁺CD68⁺ cells). (C)** **Quantitative M1 and M2 macrophage percentages of GGNs and SNs, respectively** (n = 6 patient per group)**. (D)** **Quantitative M1/M2 ratios of GGNs and SNs, respectively.**


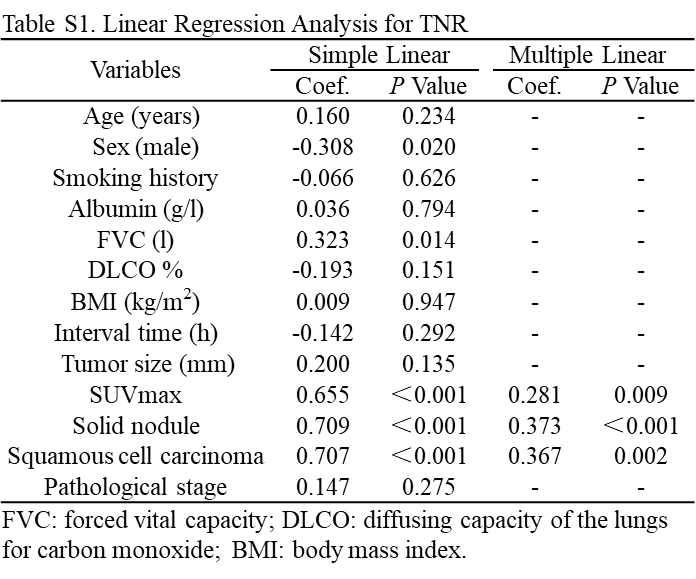

Supplement: Supplementary file 1 — Supporting Information [file ADVS-12-e04498-s002.docx]
